# Syndrome of headache with neurologic deficits and CSF lymphocytosis (HaNDL)

**DOI:** 10.1186/1129-2377-14-S1-P163

**Published:** 2013-02-21

**Authors:** K Skorobogatykh, Y Azimova, G Tabeeva

**Affiliations:** 1 1st Moscow State Medical University, Russian Federation

## Objectives

To describe a case of syndrome of headache with neurologic deficits and CSF lymphocytosis (HaNDL).

## Background

HaNDL is a rare condition with defined diagnostic criteria as outlined in section 7.8 of the second edition of the International Classification of Headache Disorders (ICHD II).

## Methods

Case Study.

## Results

Clinical presentation.

In November 2009 a 21-year-old white female experienced 5 episodes of sudden ascending left side hypoestesia, dysarthria and sensory aphasia followed by a nausea and then by severe throbbing unilateral headache. Headache was accompanying with nausea, vomiting, photo- phono and osmophobia, and was located in the right temporal area. Neurological deficit resolved in one hour, headache aborted in 8 hours. At the moment of admission she had subfebrile temperatures but she had not any focal neurological deficit or meningeal signs. Patient had not any personal history of migraine, but her mother suffered from migraineous headaches. Lumbar puncture revealed lymphocytosis (86/mm3) and slightly elevated total protein (66 mg/dL). All investigations including MRI of the brain, EEG, carotid duplex and serological tests for certain viruses and bacteria were normal. Patient had not any episode of neurological deficit or migraineous headache during 10 months follow-up. This patient fulfilled the ICHD II criteria for HaNDL syndrome. It is supposed that HaNDL syndrome could have viral, autoimmune or vascular origin or to be a rare variant of migraine with aura. There is no any specific treatment for this syndrome.

## Conclusion

HaNDL syndrome should be considered as a differential diagnosis for patients presenting with headache and neurological deficit.

